# A Shallow Water Ferrous-Hulled Shipwreck Reveals a Distinct Microbial Community

**DOI:** 10.3389/fmicb.2020.01897

**Published:** 2020-08-19

**Authors:** Kyra A. Price, Cody E. Garrison, Nathan Richards, Erin K. Field

**Affiliations:** ^1^Department of Biology, East Carolina University, Greenville, NC, United States; ^2^Program in Maritime Studies, Department of History, East Carolina University, Greenville, NC, United States

**Keywords:** microbial communities, shallow water shipwreck, iron, biocorrosion, iron-oxidizing bacteria

## Abstract

Shipwrecks act as artificial reefs and provide a solid surface in aquatic systems for many different forms of life to attach to, especially microbial communities, making them a hotspot of biogeochemical cycling. Depending on the microbial community and surrounding environment, they may either contribute to the wreck’s preservation or deterioration. Even within a single wreck, preservation and deterioration processes may vary, suggesting that the microbial community may also vary. This study aimed to identify the differences through widespread sampling of the microbial communities associated with the Pappy Lane shipwreck (NC shipwreck site #PAS0001), a shallow water ferrous-hulled shipwreck in Pamlico Sound, North Carolina to determine if there are differences across the wreck as well as from its surrounding environment. Loose shipwreck debris, drilled shipcores, surrounding sediment, and seawater samples were collected from the Pappy Lane shipwreck to characterize the microbial communities on and around the shipwreck. Results indicated that the shipwreck samples were more similar to each other than the surrounding sediment and aquatic environments suggesting they have made a specialized niche associated with the shipwreck. There were differences between the microbial community across the shipwreck, including between visibly corroded and non-corroded shipwreck debris pieces. Relative abundance estimates for neutrophilic iron-oxidizing bacteria (FeOB), an organism that may contribute to deterioration through biocorrosion, revealed they are present across the shipwreck and at highest abundance on the samples containing visible corrosion products. *Zetaproteobacteria*, a known class of marine iron-oxidizers, were also found in higher abundance on shipwreck samples with visible corrosion. A novel *Zetaproteobacteria* strain, *Mariprofundus ferrooxydans* O1, was isolated from one of the shipwreck pieces and its genome analyzed to elucidate the functional potential of the organism. In addition to iron oxidation pathways, the isolate has the genomic potential to perform carbon fixation in both high and low oxygen environments, as well as perform nitrogen fixation, contributing to the overall biogeochemical cycling of nutrients and metals in the shipwreck ecosystem. By understanding the microbial communities associated with shallow water ferrous-hulled shipwrecks, better management strategies and preservation plans can be put into place to preserve these artificial reefs and non-renewable cultural resources.

## Introduction

Shipwrecks, as solid heterogenous substrates in an aquatic ecosystem, provide microorganisms with a substrate for attachment in the form of an artificial reef (Connell, 2000; Svane and Petersen, 2001; Walker et al., 2007; Church et al., 2009). This substrate provides microbes with a rich source of nutrients, allowing others to subsequently attach and form an assemblage with dynamic community interactions. Metabolic interactions occurring between microbes and the shipwreck surface ultimately influence the structural integrity of the ship, as well as provide a hotspot of biological interactions for the surrounding environment. Coastal shipwrecks are widespread across North Carolina’s coastline and are irreplaceable resources that help to preserve cultural history. They are also home to a diverse array of microbial communities. The complex geography of North Carolina’s Outer Banks – also known as “The Graveyard of the Atlantic” – led to more than 2,000 shipwrecks since first contact by Europeans in the 16th century (Stick, 1952; Lawrence, 2008). With time, chemical and biological forces alter the structural integrity or degree of articulation of a shipwrecked watercraft. Of these biological forces, microorganisms greatly influence the overall quality of the wreck and promote the attachment of other organisms to create a community assemblage. Preservation and protection actions are enhanced by understanding the relationships between an individual wrecked ship, associated communities, and the proximal ecosystem (Mires, 2014). In an effort to achieve this, we must better understand the microbial communities associated with shipwrecks and the role they may play in its preservation and/or deterioration.

Shallow water shipwrecks may be impacted by these physical, chemical, and biological processes more than their deep ocean counterparts due to their partial exposure to the atmosphere, human interference (i.e., salvage or souvenir hunting), and the increased effects of severe weather events such as hurricanes (Damour et al., 2015). However, these effects will not be the same for every shipwreck, and thus, preservation methods must be tailored to the specific ship and environmental conditions. Even across one site or wreck, some regions are more vulnerable to deterioration while others may be better preserved. As microbes can contribute to both deterioration and preservation processes, we expect that microbial communities will show similar patterns. Yet, it remains unclear how much microbial community diversity may change across one wreck as fine-scale sample collection is limited.

Aquatic ecosystems can often be nutrient limited, and thus, attachment to surfaces such as shipwrecks can be advantageous for organic and inorganic nutrient acquisition, as well as for promoting symbiotic relationships between microbes (Connell, 2000; Svane and Petersen, 2001; Walker et al., 2007; Church et al., 2009; Haridas et al., 2017). Some microbial communities on shipwrecks, in the form of biofilms or in the surrounding sediment, play a vital role in shipwreck preservation by supporting and recruiting other microorganisms, as well as macroorganisms to attach and protect these artificial reefs (Beech and Cheung, 1995; Huggett et al., 2006). Biofilms are composed of a consortium of microorganisms, DNA, proteins, and extracellular polymeric substances that adhere to surfaces (Costerton et al., 1987, 1995). These systems consist of microbial interactions, chemical reactions, and intermingled transport processes; each of these characteristics can influence the functional role of the biofilm on any attached surface (Videla and Herrera, 2005, 2009; Zuo, 2007; Little et al., 2008). By attaching to metal structures, they eliminate access to the metal surface, but the role of the biofilm is strongly dependent on the surrounding environment (Mugge et al., 2019). These biogeochemical interactions are important to investigate and characterize because they impact the surrounding ecosystem composition and overall functions, such as nutrient and metal cycling, adding to the novelty of a shipwreck ecosystem (Mallefet et al., 2008; Yin et al., 2015; Hamdan et al., 2018; Mugge et al., 2019).

Previous studies have characterized shipwreck-associated microbial communities from a wide range of samples, including nearby sediments, surrounding water, and even metal coupons deployed near the wreck (Cullimore and Johnston, 2008; Fagervold et al., 2012; Leary et al., 2014; McBeth and Emerson, 2016; Hamdan et al., 2018; Mugge et al., 2019). Certain functional groups of microbes, such as sulfate-reducing, iron-oxidizing, iron-reducing, acid-producing, denitrifying, and heterotrophic bacteria, have been found at higher abundances near the shipwreck compared to the more distant surrounding sediment and water. Previous studies of shipwrecks in the Gulf of Mexico affected by the Deepwater Horizon oil spill found bacterial and archaeal microbiomes to be significantly different between samples taken near versus away from the shipwrecks (Hamdan et al., 2018). The *Deltaproteobacteria*, often capable of sulfate reduction and contributors to biocorrosion, were found to be the most abundant class at all sites and continued to increase with sediment depth. Other common taxa, such as *Gammaproteobacteria*, *Phycisphaerae*, and *Dehalococcoidaceae*, were also highly abundant at all sites (Hamdan et al., 2018; Salerno et al., 2018), suggesting they may be important to the microbial communities found on the shipwreck but the exact processes occurring on the surface of the shipwreck are unclear (Ravenschlag et al., 1999; Löffler et al., 2013; Robbins et al., 2016). Based on results from previous shipwreck and steel surface related studies (Cullimore and Johnston, 2008; McBeth et al., 2013; McBeth and Emerson, 2016; Hamdan et al., 2018; Garrison et al., 2019), the microbial communities found on the shipwreck in this study were hypothesized to vary in composition across the shipwreck as metal content, sample type, and sampling location across the wreck changed.

Iron-oxidizing bacteria (FeOB) and sulfate-reducing bacteria (SRB), which have been previously isolated from corroded metal infrastructure (Bermont-Bouis et al., 2007; Xu et al., 2007; Little et al., 2008; Al Abbas et al., 2013; Emerson, 2019; Garrison et al., 2019), have been shown to contribute to biocorrosion and deterioration of steel structures (Little et al., 2016; Mugge et al., 2019). Evidence of this corrosion on ferrous hulls is often in the form of corrosion products visible as rust buildup, pitting, and black precipitates on the surface. Various corrosion byproducts or waste from an organism’s metabolism such as iron oxides and/or sulfide precipitates have been sampled from different environments such as metal (Hamilton, 1985; Duan et al., 2006; Lee et al., 2013; Little et al., 2014) and wooden (Palacios et al., 2009) surfaces and their chemical and microbial community composition were analyzed. These corrosion products can be identified based on their color, orange, and black, respectively, which may indicate the presence of certain microbial community members like FeOB or SRB. This visual indication was used during sample collection in this study to identify pieces assumed to be colonized by the aforementioned corrosion-associated organisms.

While SRB have been well-studied in their association with biocorrosion of steel structures, the role of FeOB in the biocorrosion process is much less understood. They are thought to be among the primary colonizers of steel surfaces and subsequently create a microenvironment on the steel surface suitable for subsequent colonization by other organisms (McAllister et al., 2011; McBeth and Emerson, 2016; Emerson, 2019). While it is expected that they would be found associated with ferrous-hulled shipwrecks, it has yet to be demonstrated due to the lack of direct shipwreck samples that have been collected to-date. Based on previous studies that have found marine FeOB, including members of the *Zetaproteobacteria*, near shipwrecks (Cullimore and Johnston, 2008; Hamdan et al., 2018; Salerno et al., 2018) and attached to steel (McBeth et al., 2011, 2013; McBeth and Emerson, 2016; Garrison et al., 2019), it was hypothesized that they would be present on the steel surface of the Pappy Lane shipwreck. By investigating the presence and relative abundance of FeOB across the shipwreck, their contribution to corrosion and deterioration of shallow water shipwrecks can be further understood.

This study aims to identify how the microbial community structure varies across a steel-hulled shallow water shipwreck and its surrounding environment, both at the whole bacterial community level and potential biocorrosion-related FeOB, through extensive sampling. This will be a first step to determine the relationships between the shipwreck-associated microbial community and its environment, providing context for previous microbial community studies. This will also provide a comparison between shallow water shipwrecks and the well-studied deep-water shipwrecks. Ultimately, this information will help us better understand how microbes can contribute to the preservation and deterioration of a shipwreck and how to better develop preservation strategies for these historical artifacts.

## Materials and Methods

### Historical Significance

**Figure 1:**
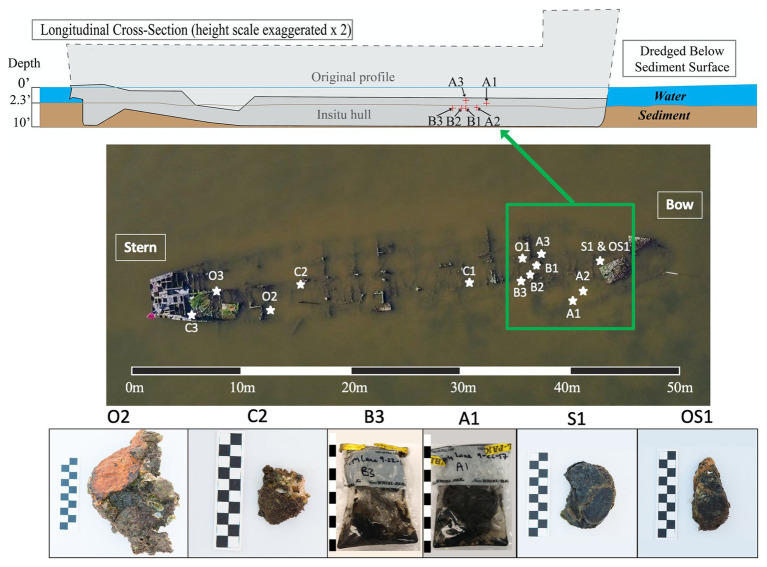
Cross-sectional schematic of the Pappy Lane Shipwreck and locations, where ship debris samples were collected based on visible corrosion (O, S, and OS samples) and non-visible corrosion products (C samples). Depth profile of shipcore samples taken by drilling from the shipwreck above the sediment line (A samples) and below the sediment line (B samples) after dredging can also be seen. A 50 m scale bar is used to display the length of the shipwreck and 10 cm scale bars are used to display the length of each piece taken (1 block = 1 cm).

This research investigation was based on the Pappy Lane shipwreck, a steel-hulled ship that lies in an area of the Pamlico Sound that has the potential to be impacted by the construction of the N.C. 12 Rodanthe Bridge (also known as the “Bonner Bridge Extension,” Rodanthe, Hatteras Island, NC). Based on strong circumstantial evidence, the wreck likely represents the remains of the USS LCS(L)(3) *123*, built as a warship in 1944 and eventually deposited in Eastern Pamlico Sound in the 1960s after a post-war career as a barge (Richards, 2018). The bow of the vessel lies at 35°36'3.19” N and 75°28'24.63” W, and the stern lies at 35°36'1.78” N and 75°28'25.34” W. The dimensions of the shipwreck were determined to be approximately 50 m in length overall, 9 m wide, and 1 m deep (Figure 1).

### Field Sampling

A total of 27 samples [loose shipwreck debris (8), drilled shipcores (6), nearby sediment (11), and surrounding seawater (2)] were collected from across the Pappy Lane shipwreck located in the Pamlico Sound in September 2017 (Figure 1; Supplementary Table S1). The shipwreck is exposed to fluctuating salinities due to season, tidal, and wind influences but retains moderate to high salinity due to its location (Jia and Li, 2012). The salinity and water temperature of the site on the first day were 17.9 ppt and 23.5°C, respectively, and during the second trip, these increased slightly to 26.3 ppt and 24.9°C, respectively. All loose shipwreck debris, nearby sediment, and seawater samples were collected on the first day while drilled shipcores were collected on the second day.

Ferrous shipwreck debris samples with visible corrosion products formed on the surface and samples without visible corrosion products were collected for analysis (Figure 1). Debris associated with the shipwreck were collected along the length of the shipwreck, submerged within the water column but not buried. These samples were identified based on the color of chemical constituents found on the shipwreck indicating likely biocorrosion products such as black sulfides (S), orange iron oxides (O), or both (OS). Control samples (C) with no visible corrosion products were also collected and contained shipwreck metal combined with remnants of oyster shell attached. Drilled shipcore samples were also collected using a hammer drill both above (A) and just below (B) the sediment, where the sediment had been dredged immediately before (Figure 1). All samples were collected with gloves and transported back to the lab on ice in pre-sterilized containers with enough *in-situ* water (~300 ml) to catch biological material from the piece of shipwreck during processing in the lab. The act of picking up shipwreck pieces out of the water may have disturbed the attached biofilm community. Sediment samples were collected in 50 ml conical centrifuge tubes from the same location as the shipwreck samples, as well as at four additional locations that were 9.75 m from the midline of the wreck. Surface water was collected in a pre-sterilized 3-gallon Nalgene carboys approximately 6 m away from the shipwreck location so that any organic matter kicked up from the sampling activity was avoided.

### Sample Processing

Biological material was scraped from the external surface of each shipwreck piece – the visibly corroded debris, control debris, and drilled shipcores – and subsequently collected in a 50 ml conical tube. Before centrifugation, the collected material was used to inoculate plates for a most probable number (MPN) growth study (see details below; Garrison, et al., 2019). After inoculating cultures, the remaining material was centrifuged at 4,000 × *g* for 10 min in order to concentrate material for DNA extraction. Supernatant in the tube was poured off and the remaining pellet was stored at −80°C until used in DNA extractions. Surface water was filtered using a 0.22 μm polyethersulfone filter; the filter was aseptically removed and stored at −80°C along with the sediment samples for subsequent DNA extraction.

### Quantification of FeOB

Orange iron oxide ferrous debris samples were selected to culture in enrichment media specific for FeOB (Field et al., 2015; Garrison et al., 2019) to determine the presence and relative abundance of all possible neutrophilic microaerophilic FeOB in each sample. FeOB enrichment media in the form of estuary media (EM) was used similar to previous studies. Briefly, 1 L of EM consists of: 13.75 g NaCl, 2.69 g MgCl_2_•6H_2_O, 3.49 g MgSO_4_•7H_2_O, 0.36 g KCl, 0.75 g CaCl_2_•2H_2_O, 1 g NH_4_Cl, 0.05 g KH_2_PO_4_, 1.95 g MES, and 0.42 g NaHCO_3_. The media were prepared and autoclaved; then, once cooled, vitamins and trace minerals (ATCC MD-VS and MD-TMS) were added according to manufacturer’s recipe (1 ml per 1 L). EM were used in these growth studies due to the salinity of the sampling site being greater than 5 ppt. Previous studies have shown that freshwater FeOB can tolerate up to 5 ppt and marine FeOB can tolerate down to 5 ppt; therefore, a threshold of 5 ppt was used to determine which media type qualified for FeOB enrichment (McBeth et al., 2013; Chiu et al., 2017).

A MPN method was used by incorporating a series of replicates and dilutions to estimate the presence and relative abundance of FeOB. Enrichment cultures allowed estimations of FeOB relative abundance without the bias of measuring only certain species or classes of FeOB (i.e., *Zetaproteobacteria*) such as more molecular specific methods, e.g., quantitative PCR. The biological material removed from ferrous debris pieces was used as inoculum; 1.5 ml of the sample, prior to centrifuging for DNA extractions, was inoculated into petri dishes containing 13.5 ml media for a total plate volume of 15 ml. Following the procedure described in Garrison et al. (2019), each sample was plated to three replicates with four dilutions of each replicate (12 plates total for each shipwreck piece) for MPN estimates. For each dilution transfer, the previous plate was randomly mixed, and the material was randomly pipetted. Five milligrams of zero valent iron mesh (200 μm) was then added to each plate as a highly bioavailable source of iron for any FeOB which could have been present in the sample (Garrison et al., 2019).

After inoculation, the MPN enrichment cultures were stored with BD GasPak EZ Campy Container Systems to create microaerobic conditions (~1% O_2_ headspace) during incubation. The containers were sealed and incubated at 20°C for 21 days to ensure optimum growth of FeOB cells that could have been present (Emerson and Floyd, 2005; Garrison et al., 2019). An incubation temperature of 20°C was chosen for all sample cultures to avoid slow growth rates, which could have affected the results after 21 days, as well as to be able to align with previously reported optimal growth temperatures in the literature (Chiu et al., 2017; Garrison et al., 2019). FeOB growth for each plate was determined based on the production of biological iron oxides and then, if necessary, confirmed with both light microscopy for distinctive helical iron oxide morphologies and fluorescence microscopy for distinctive bean-shaped cells attached to the iron oxides, and additional confirmation was achieved by subsequently transferring the potential positive enrichment culture to a new enrichment plate. Positive growth by FeOB appears as very distinctive suspended flocculant orange clumps, which are biologically produced iron oxide stalks intertwined together (Little et al., 2014; Garrison et al., 2019). An MPN calculator (Curiale, 2012) was used to estimate abundance based on which plates out of the set for each sample exhibited FeOB growth after the incubation period.

The MPN results were normalized to the surface area of their corresponding shipwreck piece. Surface area of each piece was determined using ImageJ 1.51 (Schneider et al., 2012). After loading the photograph into ImageJ, the scale of the image was set by including a ruler in each photograph, followed by converting the length in centimeters to pixels using the set scale feature of ImageJ. The outline of the shipwreck piece was traced using the line tool, and the surface area was determined using the measure area tool. MPN abundance data were used to determine the presence or absence of FeOB for each sample, their relative abundance, and their distribution across the shipwreck.

### Quantitative PCR

Due to the high salinity of the site and identification of the isolate as a *Zetaproteobacteria*, a marine FeOB, quantitative PCR was used to determine the presence and relative abundance of *Zetaproteobacteria* across the ship debris pieces. CFX Connect™ Real-Time PCR Detection System and SsoAdvanced™ Universal SYBR® Green Supermix was used to determine the relative abundance of *Zetaproteobacteria* using the primers Zeta672F (5'-CGG AAT TCC GTG TGT AGC AGT-3') and Zeta837R (5'-GCC ACW GYA GGG GTC GAT ACC-3'; Kato et al., 2009). Following the protocol supplied in the SsoAdvanced™ Universal SYBR® Green Supermix instruction manual (2013), each PCR reaction mix contained 10 μl (2X) Supermix, 1 μl of each (10 μM) forward and reverse primers, 2 μl (~50 ng) DNA template, and the rest of the volume of nuclease-free water (6 μl), so each reaction was 20 μl total. PCR conditions were 1 cycle of 3 min at 98°C, followed by 35 cycles of 15 s at 95°C and 30 s at 60°C. The percent of *Zetaproteobacteria* to total DNA in each sample was determined using the Biorad CFX Maestro Software v1.1 and the average relative abundance was calculated and graphically displayed using R (R Core Team, 2013).

### Species Isolation and Sequencing

Three successful dilutions-to-extinction in FeOB enrichment media, performed the same as the MPN media method described above, led to the isolation of a marine *Zetaproteobacteria* FeOB referred to as *Mariprofundus ferrooxydans* strain O1 – a marine FeOB strain from the O1 shipwreck piece – based on phylogeny and whole genome comparisons with other cultivated *Zetaproteobacteria*. The pure culture was confirmed using epifluorescence microscopy to identify the presence of a single cell morphology, followed by inoculation onto standardized nutrient agar [9 g/L nutrient broth (Remel) and 15 g/L bacteriological agar (VWR International, LLC.,)] to confirm the absence of contaminating heterotrophs.

DNA from the isolate *Zetaproteobacteria* strain was extracted using 250 mg of sample material and the DNeasy PowerSoil Kit (Qiagen, Inc.,). DNA was subsequently PCR amplified using universal 16S primers 8F and 1492R (Lane, 1991; Tuer et al., 1999). PCR products were purified using a QIAquick PCR purification kit (Qiagen, Inc.,). The 16S ribosomal RNA (rRNA) gene was sequenced *via* Sanger sequencing by GeneWiz (South Plainfield, NJ) to identify the isolate. The sequence data were aligned to create a consensus sequence and cleaned using Sequencher® (Gene Codes Corporation, Ann Arbor, MI). The aligned 16S rRNA gene sequence was then imported into BLAST (Altschul et al., 1990) to identify which species were close neighbors. The aligned 16S rRNA gene sequence was also imported into the ZetaHunter program (McAllister et al., 2018) to identify which *Zetaproteobacteria* OTU it belonged to.

The entire genome of the isolate was sequenced by CGEB-IMR© (Dalhousie University, Halifax, NS, Canada) using Illumina MiSeq. The paired-end reads were trimmed and filtered using Trimmomatic (Bolger et al., 2014) and FastQC (Andrews, 2010), assembled using SPAdes (Bankevich et al., 2012) and annotated using RASTtk (Aziz et al., 2008; Overbeek et al., 2013; Brettin et al., 2015). Analysis of the genome was performed using RAST and the genome size and completeness was determined using CheckM (Aziz et al., 2008; Overbeek et al., 2013; Parks et al., 2015). Maximum likelihood phylogenetic trees for 16S rRNA gene comparisons with other similar bacteria lineages were constructed using MEGAX (Kumar et al., 2018) using the Tamura-Nei nucleotide substitution model for 16S rRNA with 1,000 bootstrap iterations.

### Community Analysis

In order to characterize the microbial communities present on and around the shipwreck, 250 mg of sample material was extracted for DNA from all samples collected using a MoBio DNeasy PowerSoil Kit (Qiagen, Inc.,), and those with high quality DNA were subsequently sent to Dalhousie University Integrated Microbiome Resource (IMR; imr.bio) for 16S rRNA gene amplicon sequencing (V4-V5 region). DNA extractions from drilled shipcore samples A2, B1, and B2 did not yield enough DNA for amplicon sequencing after three repeated attempts and thus were removed from the downstream microbial community analyses. 16S amplicons were generated using improved internal transcribed spacer marker gene primers (Walters et al., 2015) according to the protocol in Comeau et al. (2017) and sequenced using 300 + 300-bp PE v3 chemistry and on an Illumina MiSeq. Sequence data from loose shipwreck debris O3, sediment samples 9.75 m from the midline of the ship, O1, S1, and C3, and drilled shipcore A3 were not complete and therefore were removed from the subsequent microbial analysis. Microbial community data were processed and analyzed using mothur v1.41.3 (Schloss et al., 2009) and associated curation pipeline (Kozich et al., 2013). The resulting community analysis data were manipulated using non-metric multidimensional scaling (NMDS) in R using vegan and ggplot2 packages (R Core Team, 2013). Within the vegan package, metaMDS function with Bray-Curtis dissimilarity method was used to construct NMDS plots, ANOSIM function was used to determine significant differences in microbial community composition between sample types, and SIMPER function was used to determine which taxa specifically were most influential in causing those community differences between sample types.

### NCBI Accession Numbers

The 16S amplicon sequence data can be found in the Short Read Archive (SRA) database under the accession numbers SRR12148233–SRR12148251. The GenBank accession number for the 16S rRNA gene sequence of *M. ferrooxydans* O1 is MT238205. The NCBI accession number for *M. ferrooxydans* O1 whole-genome sequence is JAAVJJ000000000. Specific genes of interest from *M. ferrooxydans* O1 discussed in the results were also deposited in GenBank and can be found in the text below. The accession number for the whole project is PRJNA614966.

## Results

**Figure 2:**
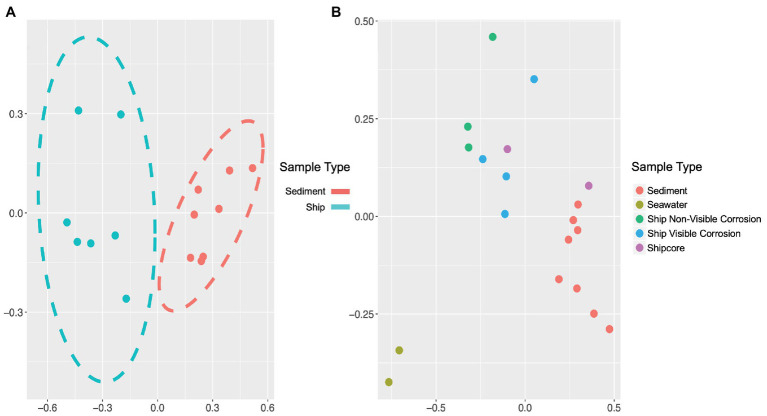
Non-metric multidimensional scaling (NMDS) plots of 16S ribosomal RNA (rRNA) gene amplicon sequence data, which demonstrate differences in microbial community composition based on sample type or environment. Microbial community composition was distinct when comparing **(A)** visibly and non-visibly corroded samples to sediment samples (ANOSIM test: *R* = 0.86, *p* = 0.0004) and **(B)** when including surrounding seawater and shipcore samples (ANOSIM test: *R* = 0.75, *p* = 0.0001). Stress values for plots A and B are 0.058 and 0.072, respectively.

**Figure 3:**
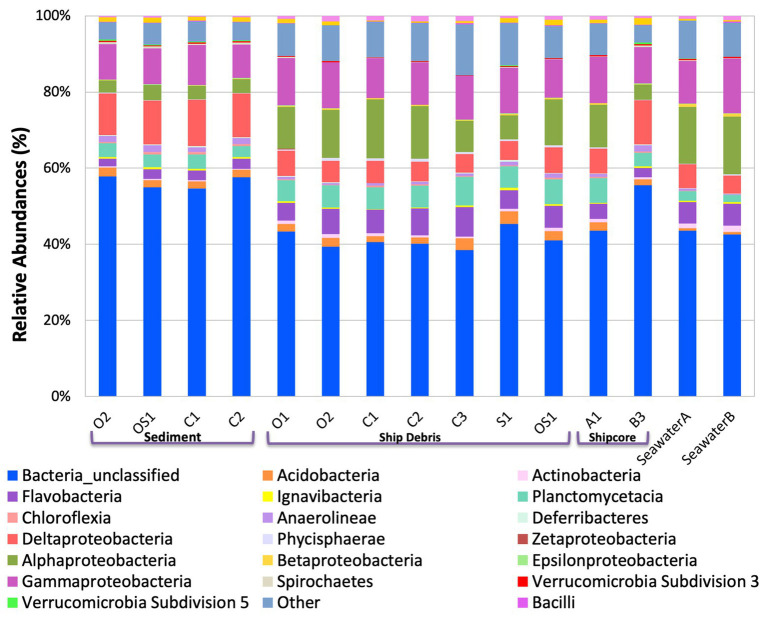
Class level taxa plot of the shipwreck and surrounding environment microbial community composition present on each sample. Relative abundance calculated based on the percent of total class-level OTUs per sample.

The microbial community composition and OTU relative abundances at the shipwreck site differed between sediment and ship samples (Figure 2A; ANOSIM test: *R* = 0.86, *p* = 0.0004) and between all sample types, including visibly corroded ship debris, non-visibly corroded ship debris, sediment, seawater, and shipcore samples (Figure 2B; ANOSIM test: *R* = 0.75, *p* = 0.0001). There were notable differences in microbial community composition at both phyla and class levels between the types of samples collected (Figure 3; Supplementary Figure S1). The taxa that contributed the most to differences seen in community composition between ship and sediment samples at the phyla level were unclassified bacteria (SIMPER analysis: 55.8%) and *Proteobacteria* (21.7%), and at the class level were unclassified bacteria (45.8%), *Deltaproteobacteria* (13.8%), *Alphaproteobacteria* (5.8%), and *Gammaproteobacteria* (5.7%). At the phyla level, *Proteobacteria* were more abundant on the ship debris while the unclassified bacteria were more abundant in the sediment. At the class level, both *Alphaproteobacteria* and *Gammaproteobacteria* were more abundant on the ship debris while the unclassified bacteria and *Deltaproteobacteria* were more abundant in the sediment. The taxa that contributed the most to differences between visibly corroded and non-visibly corroded ship debris at the phyla level were *Proteobacteria* (SIMPER analysis: 43.2%), unclassified bacteria (22.5%), and *Bacteroidetes* (12.9%) and at the class level were unclassified bacteria (46.3%), *Alphaproteobacteria* (15.0%), *Gammaproteobacteria* (10.3%), *Deltaproteobacteria* (7.4%), and *Planctomycetia* (6.0%). At the phyla level, unclassified bacteria and *Proteobacteria* were more abundant on the visibly corroded ship debris while *Bacteroidetes* were more abundant on the non-visibly corroded ship debris. At the class level, *Gammaproteobacteria* and *Deltaprotoebacteria* were more abundant on the visibly corroded debris while *Alphaproteobacteria* and *Planctomycetia* were more abundant on the non-visibly corroded debris.

The variability observed in microbial community composition between ship debris and sediment or seawater environments suggests that there may be niche partitioning by some taxa associated with the shipwreck (Figure 3; Supplementary Figure S1). Of the classes previously identified in higher proportions near shipwrecks (*Gammaproteobacteria*, *Phycisphaerae*, and *Dehalococcoidaceae*; Hamdan et al., 2018; Salerno et al., 2018), only *Phyciscphaerae* were found in higher proportion on the shipwreck debris compared to the other sample types. The shipwreck debris samples also had higher proportions of *Bacteroidetes*, *Planctomycetes*, and *Verrucomicrobia* compared to other sample types. The classes with higher proportions on the shipwreck debris compared to other sample types were *Alphaproteobacteria*, *Flavobacteria*, *Planctomycetacia*, and *Verrucomicrobiae*. All of these taxa were also found at a higher proportion in the above-ground drilled samples and seawater samples compared to the sediment and below-ground drilled piece.

Interestingly, the microbial community of the drilled shipcore sample from below the sediment line was more similar to the sediment samples than the above-ground shipcore samples or debris samples. A higher proportion of the phyla *Chloroflexi*, *Deferribacteres*, and *Firmicutes* and the classes *Anaerolinae*, *Chloroflexi*, *Clostridia*, and *Deltaproteobacteria* were found on the below-the-sediment-line drilled shipcore samples than on the shipwreck debris. It is worth noting that after sample collection, it was determined that the drilled samples came from regions that may have stored hydrocarbons at one time and the presence of *Chloroflexi*, known hydrocarbon degraders (Dombrowski et al., 2017), may be indicative of this.

**Figure 4:**
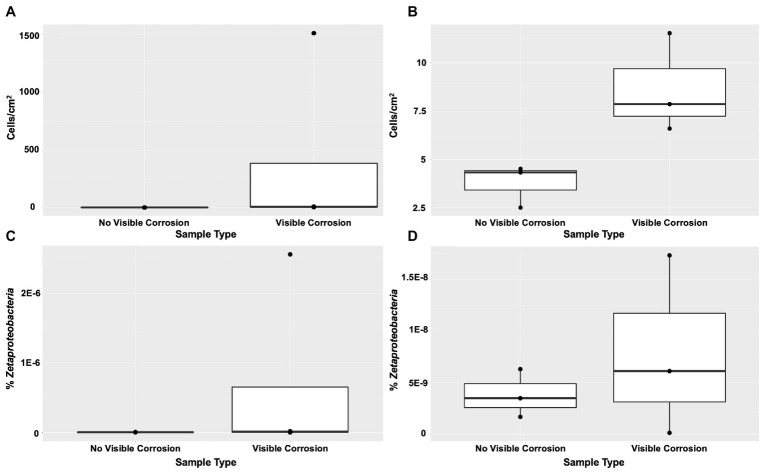
Iron-oxidizing bacteria (FeOB) were found in all shipwreck samples with greatest relative abundance in samples with visible corrosion based on a most probable number (MPN) method **(A)** with inclusion of outlier sample O1 (Mann-Whitney U test: *W* = 12, *p* = 0.057) and **(B)** without inclusion of outlier sample O1 (*W* = 9, *p* = 0.1). Proportion of *Zetaproteobacteria* out of total community/0.25 g of material collected was shown by quantitative PCR **(C)** with inclusion of outlier sample O1 (*W* = 8, *p* > 0.05) and **(D)** without inclusion of outlier sample O1 (*W* = 5, *p* > 0.05).

FeOB were found widespread across the shipwreck as there was positive growth on all MPN plates, with higher relative abundance of growth (cells/cm^2^) on the samples that contained visible corrosion products (Figures 4A,B). The highest FeOB relative abundance was on the O1 shipwreck debris piece with >1,511 cells/cm^2^ compared to ~4 cells/cm^2^ on the control samples. The O1 debris sample was orders of magnitude greater than all other samples with the average FeOB cell counts, including sample O1 was 384 cells/cm^2^ ± 751 and excluding O1 was 9 cells/cm^2^ ± 2.6. When including O1 debris sample, there was a greater significant difference in FeOB relative abundance between ship debris with or without visible corrosion (Mann-Whitney U test: *W* = 12, *p* = 0.057) compared to exclusion of sample O1 (*W* = 9, *p* = 0.1; Figures 4A,B). Exclusion of sample O1, however, shows that visibly corroded samples still exhibited higher relative abundances of FeOB overall compared to visibly non-corroded ship debris. Sample O1 may have had higher relative abundance of FeOB cells due to an elemental composition of the debris piece more favorable to FeOB growth, but the fine-scale metal content of this wreck is unknown.

*Zetaproteobacteria*, a known marine FeOB class, were also found widespread across the shipwreck based on qPCR analyses (Figures 4C,D). There were higher proportions of *Zetaproteobacteria* on shipwreck samples with visible corrosion products than those without, again supporting the same trend seen from the MPN results. Additionally, there was more variation in the proportions of *Zetaproteobacteria* among those samples with visible corrosion products than those without, suggesting that there are multiple factors affecting their abundance such as iron content, exposure to oxygen, and/or the presence of other community members. The highest proportion of *Zetaproteobacteria* was found on the Sample O1 shipwreck debris piece (2.6 × 10^−6^% *Zetaproteobacteria* out of total community/0.25 g material collected) and was three orders of magnitude higher than all other samples. There was no significant difference in *Zetaproteobacteria* proportion between shipwreck samples with or without visible corrosion (Including O1: Mann-Whitney U test: *W* = 8, *p* > 0.05; Excluding O1: *W* = 5, *p* > 0.05; Figures 4C,D). The average proportion of *Zetaproteobacteria* in the community including sample O1 was 6.5 × 10^−7^ ± 1.3 × 10^−6^% and excluding O1 was 7.9 × 10^−9^ ± 8.7 × 10^−9^%.

**Figure 5:**
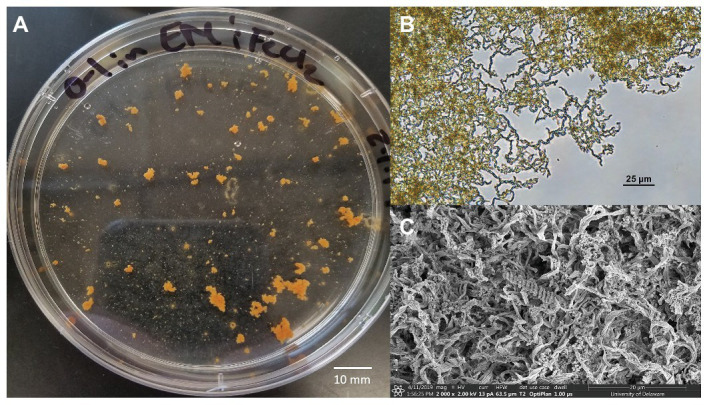
*Mariprofundus ferrooxydans* O1 **(A)** grown in estuary media (EM) culture media producing distinctive, suspended flocculant orange clumps in the form of biologically produced iron oxides intertwined into braided stalks, as seen under **(B)** Bright-field microscopy and **(C)** scanning electron microscopy.

A novel *Zetaproteobacteria* strain was successfully isolated from the O1 shipwreck debris piece through dilution-to-extinction cultures and microscopy. This isolate was identified as *M. ferrooxydans* species based on 16S rRNA gene analysis and whole genome comparisons with other known *Mariprofundus* strains. This isolate grew in EM culture media and formed distinctive, suspended flocculant orange clumps (Figure 5) and produced iron oxyhydroxides intertwined into braided stalks, typical of *Mariprofundus* spp. (Singer et al., 2011; Mumford et al., 2016). The draft genome sequence of *M. ferrooxydans* O1 consists of 21 contigs. It has a genome size of 2,760,518 bp, a GC content of 54.2%, and 2,821 protein-coding sequences and is 99% complete with 0% redundancy and 0% strain heterogeneity. The 16S rRNA BLAST results of Strain O1 identified the most closely related organisms as marine iron-oxidizing *Mariprofundus* strains within *Zetaproteobacteria* OTU11 (ZOTU11; Supplementary Figure S2): *Mariprofundus* sp. SC-2 [99.66% average nucleotide identity (ANI)], *M. ferrooxydans* M34 (99.64% ANI), *M. ferrooxydans* JV-1 (99.31% ANI), *M. ferrooxydans* PV-1 (99.28% ANI), and *Mariprofundus* sp. EKF_M39 (96.50% ANI) (Supplementary Figure S2; Yoon et al., 2017).

Further analysis of the *M. ferrooxydans* O1 genome suggests that this organism contains both Type I and Type II ribulose-1,5-bisphosphate carboxylase/oxygenase (RubisCO) genes used in carbon fixation (RubisCO Type I GenBank Accession # MT709282-MT709283; RubisCO Type II GenBank Accession # MT709284). The amino acid sequences of both the large and small subunit for Type I, as well as the amino acid sequence for Type II, were most similar to *M. ferrooxydans* PV-1 with 100% identity. Using the cyc2 sequence taken from *M. ferrooxydans* PV-1, strain O1 was found to contain a hypothetical protein (GenBank Accession # MT709281) possibly involved in iron oxidation with an e-value of 0.0 and 83% identity at the gene level (McAllister et al., 2020).

Through comparative genomics of *Zetaproteobacteria* isolates within ZOTU11, the minimum set of *nif* genes (*nifHDKENB*) required to perform nitrogen fixation were identified in the strain O1 genome and found to be highly conserved between the strain O1, *M. ferrooxydans* M-34, and *Mariprofundus* sp. EKF-M39 (Supplementary Tables S2, S3; Supplementary Figure S2). The *nifH* gene sequence found in strain O1 (Genbank accession # MT709280) encodes for dinitrogenase reductase, which is an essential component of the nitrogenase enzyme (Arcondéguy et al., 2001; Johnston et al., 2005; Dos Santos et al., 2012; Kato et al., 2015b), and is a commonly used marker gene for nitrogen fixation. This gene along with others that make up the nitrogenase enzyme were used to analyze the genomes of FeOB isolates to determine if they have the genomic potential to perform nitrogen fixation. Nitrogen fixation capabilities have been confirmed in isolates *M. ferrooxydans* M34 and *Mariprofundus* sp. EKF-M39 (Field et al., 2015). These genes could not be found in either *M. ferrooxydans* PV-1 or JV-1, but it is possible that these genes are missing, as their genomes are not complete. Nitrogen fixation genes have also been found in *Mariprofundus* sp. DIS1, as well as in freshwater FeOB isolates *Sideroxydans lithotrophicus* ES-1 and *Ferriphaselus amnicola* OYT1 (Kato et al., 2015b).

## Discussion

The microbial communities were distinctly different across each type of sample collected (i.e., shipwreck debris, drilled shipcores, sediment, and seawater). There were differences in the microbial communities even between visibly corroded and non-visibly corroded ship debris pieces, indicating that the surface material the microbes were attached to influences the community composition. Each sample type contained community members that likely contribute to the biogeochemical cycling of nutrients and metals in the shipwreck ecosystem. According to the NMDS models created for the different sample types, there was little variation in the microbial communities within the same sample type, suggesting that the microbial communities attached to similar materials have similar dominant microbial community members, but may vary in rare taxa. Certain taxonomic classes such as *Flavobacteria*, *Phycisphaerae*, and *Planctomycetaciae* that are usually found in a marine environment were present in the shipwreck debris communities at higher abundance than the seawater samples. These organisms have also been previously found in the sediment near well-studied deep-water shipwrecks, which may suggest that these organisms preferentially attached to the shipwreck surface (Hamdan et al., 2018). Overall, the variability in microbial community composition across the shipwreck may be due to differences in the chemical composition of the ship itself or due to small-scale variability in the environment surrounding the shipwreck.

These distinct, yet consistent, microbial communities associated with the shipwreck may have important implications for biogeochemical cycling in the shipwreck environment. For example, nitrogen cycling may be widespread in the microbial communities associated with the shipwreck surface *via Zetaproteobacteria* that may fix nitrogen and *Planctomycetes* that were highly abundant on the shipwreck and are known to perform ammonia oxidation, which contributes to an increase in dinitrogen gas. An abundance of this unavailable nitrogen in the ecosystem could provide a nitrogen source for organisms capable of fixing dinitrogen gas leading to a symbiotic relationship between these community members.

Microbial communities in the sediment and on the below-ground drilled sample were more similar to each other than to other sample communities, including the above-ground drilled samples, indicating that the *in situ* environmental conditions and the proximity of nearby communities were likely important factors in determining community composition. The microbial community members that were more abundant in the sediment samples and the below-ground drilled sample were *Anaerolineae*, *Deferribacteres*, *Deltaproteobacteria*, *Chloroflexia*, and *Clostridia*, are typically found in anaerobic environments, and are capable of performing functions such as anaerobic ammonia oxidation, sulfate-reduction, and hydrocarbon degradation. A well-characterized functional metabolism of the phylum *Chloroflexi* is hydrocarbon degradation (Dombrowski et al., 2017). The higher abundance of *Chloroflexi* in the sediment samples and below-ground shipcore sample may suggest the presence of hydrocarbons in the sediment likely being released from a fuel tank within the shipwreck itself. *Deferribacteres* phylum has been previously characterized to use a variety of electron donors and acceptors in various metabolic pathways such as iron (III)-reduction, sulfate-reduction, and nitrate-reduction, which allow these microorganisms to inhabit a variety of niches and provide many ecosystem processes to their surrounding environment (Tamazawa et al., 2017). The high abundance of *Deferribacteres* in these samples suggests that these compounds are likely concentrated in the sediment surrounding the shipwreck and may be coming from the microorganisms that use different parts of the shipwreck as electron donors. Additionally, *Deltaproteobacteria*, which are strict anaerobes known to reduce iron (III) and sulfate (Slobodkina et al., 2012; Kato et al., 2015a), were found in all samples, and more specifically at higher abundances within both sediment and below-ground drilled sample. This result was similar to that of Hamdan et al. (2018), where *Deltaproteobacteria* were found in all sediment samples taken and were the more dominant community members as sediment depth increased.

While the overall dominant microbial community members across the shipwreck were similar, the rare taxa, such as the potential biocorrosion-related microorganisms, including the *Deltaproteobacteria* and *Zetaproteobacteria*, may be correlated with specific regions of the shipwreck or surrounding environment. Therefore, monitoring their location across these wrecks may be critical in designing better preservation methods. The specific metal content of this steel-hulled shipwreck remains unknown, but future studies in which the metal content is well-defined will aid in narrowing down the characteristics of these wrecks that may correspond to these organisms’ growth niches.

The findings of this study also support the widespread presence of FeOB directly on the surface of a deteriorating ferrous-hulled shipwreck. FeOB abundances were highest where visible corrosion was observed but varied across the shipwreck. Of the FeOB present, the marine iron-oxidizers were found at a low abundance within the microbial community based on 16S rRNA gene amplicon sequencing. FeOB may also exhibit a multitude of metabolisms alongside iron oxidation such as carbon and nitrogen fixation, allowing them to fulfill less energetically favorable niches for metabolisms that are key steps in the biogeochemical cycling of these elements in all environments. Previous studies analyzing the genomes of FeOB isolates found a combination of both types of the RubisCO genes required for carbon fixation in different environments, as well as the minimum set of *nif* genes (*nifHDKENB*) required to perform nitrogen fixation (Field et al., 2015). Type I RubisCO is used by organisms in high oxygen, low carbon dioxide environments while Type II is used in low oxygen, high carbon dioxide environments. Since the *Zetaproteobacteria* isolate was found to contain both types of RubisCO genes, it may be able to perform carbon fixation under fluctuating environmental conditions that they may be exposed to on the surface of these artificial reefs.

The *M. ferrooxydans* O1 described herein, as well as two other isolates in ZOTU11 – *Mariprofundus* sp. M34 and *Mariprofundus* sp. EKF-M39 – have the genomic potential to carry out nitrogen fixation, suggesting that this metabolic function may be more widespread in this group than previously described. The nitrogenase reductase subunit NifH is commonly used as a functional protein marker for nitrogen fixation (Arcondéguy et al., 2001) as it is the electron delivering iron protein of the nitrogenase complex and nitrogen fixation cannot be performed without it. This subunit, along with others that makeup the minimum set of *nif* genes (*nifHDKENB*) required to perform nitrogen fixation, were identified in three of the ZOTU11 isolates’ genomes and found to be highly conserved between them. Some of these subunits were also found in three neutrophilic FeOB that are not within ZOTU11 – *Mariprofundus* sp. DIS1, *Sideroxydans lithotrophicus* ES-1, and *Ferriphaselus amnicola* OYT1 (Arcondéguy et al., 2001; Johnston et al., 2005; Dos Santos et al., 2012; Kato et al., 2015b). The shipwreck ecosystem is a nitrogen limited environment where organisms that fix atmospheric nitrogen to a biologically available form, such as this FeOB isolate, have been known to form symbiotic relationships with other organisms in the attached assemblage (Fiore et al., 2010). While nitrogen fixation has been confirmed in *M. ferrooxydans* M34 and *Mariprofundus* sp. EKF-M39 (Field et al., 2015), it has yet to be confirmed in *M. ferrooxydans* O1 and should be further investigated.

The results from this study provide fundamental knowledge of the microbial communities associated with the shallow water ferrous-hulled Pappy Lane shipwreck, which can be used to aid in resource management and conservation of shallow water shipwrecks and other irreplaceable artifacts that have fallen victim to Earth’s watery depths. The variation in microbial community composition between sample types suggests that the communities are correlated with sample composition and surrounding environment, and this trend may also be seen in other shallow water shipwrecks. The microbial community members attached to the wreck likely contribute to nitrogen, carbon, sulfur, and iron cycling within the shipwreck environment. While this is expected of any biofilm community, in regions where a large number of wrecks are found they may be large contributors to biogeochemical cycling in coastal environments.

There are many variables that affect shipwrecks and their rates of degradation but the results presented here suggest that there are important ecosystem benefits correlated to the microbiome of the wreck. There may not be a shipwreck degradation/corrosion “model” that can be broadly applied to all wrecks, but the distinct microbiome likely reflects the wreck’s material composition as well as the environment surrounding it. Characterizing the microbial community found on the Pappy Lane shipwreck has provided insight into the ecosystem supporting coastal shipwrecks not only in North Carolina but across all coastlines. As we continue to understand more about the microbial ecosystem of shipwrecks, we can begin to apply the most appropriate methods for preserving these submerged cultural resources.

## Data Availability Statement

The datasets presented in this study can be found in online repositories. The names of the repository/repositories and accession number(s) can be found in the article/Supplementary Material.

## Author Contributions

KP, CG, and EF contributed to the experimental design. KP and CG conducted laboratory experiments and microbial community analyses. NR coordinated and oversaw field sampling efforts. All authors contributed to sample collection, data analysis, manuscript revisions, as well as have read and approved the submitted version.

### Conflict of Interest

The authors declare that the research was conducted in the absence of any commercial or financial relationships that could be construed as a potential conflict of interest.
