# Supplementary material for: A Shallow Water Ferrous-Hulled Shipwreck Reveals a Distinct Microbial Community
Source: Front Microbiol. 2020 Aug 19;11:1897. doi: 10.3389/fmicb.2020.01897 (PMC7466744; doi:10.3389/fmicb.2020.01897)
Supplement: Supplementary file 1 [file Data_Sheet_1.PDF]

Supplementary Material

A Shallow Water Ferrous-Hulled Shipwreck Reveals a Distinct Microbial Community

Kyra A. Price, Cody E. Garrison, Nathan Richards, Erin K. Field

1 Supplementary Tables

Supplementary Table S1. Description of Samples Collected from the Pappy Lane Shipwreck, with visible corrosion or no visible corrosion.

Sample ID	Sample Type	Sample Description	Depth (m)
O1	Orange loose debris	Two pieces, one smaller than the other. Both have potential corrosion products of orange iron oxides covering surface	0.67
O2	Orange loose debris	Largest piece obtained, with some parts containing oyster shells. Piece appear to have visible corrosion products of orange iron oxides covering surface	0.91
O3	Orange loose debris	Medium sized piece, half is thought to be oyster shell. Piece appear to have visible corrosion products of orange iron oxides covering surface	0.67
OB1	Orange & Black loose debris	Two pieces, one is half the size of the other, and both are thought to contain oyster shells. Both have visible corrosion products of orange iron oxides and black hydrogen sulfide precipitates covering surface	0.76
B1	Black loose debris	Piece has visible corrosion product of black hydrogen sulfide precipitate covering surface	0.76
C1	Control loose debris	Piece appears to be covered in biological material but does not have visible corrosion products. Piece is medium-sized, contains oyster shell.	0.79
C2	Control loose debris	Piece appears to be covered in biological material but does not have visible corrosion products. Piece is small and contains oyster shell.	0.79
C3	Control loose debris	Piece is relatively large and appears to be covered in biological material but does not have visible corrosion products.	1.14
A1	Above ground drilled piece	Above the sediment line, an underwater drill with a hole saw drill bit was used to remove a donut-shaped piece of shipwreck. Biological material and orange corrosion could be seen on outer side of metal piece.	0.85
A2	Above ground drilled piece	Above the sediment line, an underwater drill with a hole saw drill bit was used to remove a donut-shaped piece of shipwreck. Biological material and orange corrosion could be seen on outer side of metal piece.	0.67

A3	Above ground drilled piece	Above the sediment line, an underwater drill with a hole saw drill bit was used to remove a donut-shaped piece of shipwreck. Biological material and orange corrosion could be seen on outer side of metal piece.	0.61
B1	Below ground drilled piece	Below the sediment line, an underwater drill with a hole saw drill bit was used to remove a donut-shaped piece of shipwreck. No visible biological material or corrosion seen on piece.	0.73
B2	Below ground drilled piece	Below the sediment line, an underwater drill with a hole saw drill bit was used to remove a donut-shaped piece of shipwreck. No visible biological material or corrosion seen on piece.	0.73
B3	Below ground drilled piece	Below the sediment line, an underwater drill with a hole saw drill bit was used to remove a two donut-shaped pieces of shipwreck. No visible biological material or corrosion seen on piece.	0.73

Supplementary Table S2. *Zetaproteobacteria* Isolates ZOTU11 Showing the Presence (check mark) or Absence (X's) of Nitrogenase Subunit Genes Found in each *Zetaproteobacteria* Genome.

**Function**	**Nitrogenase Subunits**	***Mariprofundus ferrooxydans* O1**	***Mariprofundus* sp. M34**	***Mariprofundus* sp. EKF_M39**
Nitrogenase reductase Fe protein	*nifH*	✓	✓	✓
Positive regulator of *nif* transcription	*nifA*	✓	✓	✓
Nitrogenase molybdenum-iron protein alpha chain	*nifD*	✓	✓	✓
Nitrogenase molybdenum-iron protein beta chain	*nifK*	✓	✓	✓
Acivation of the Fe protein	*nifM*	✓	✓	✓
FeMoCo cofactor biosynthesis	*nifE*	✓	✓	✓
FeMoCo cofactor biosynthesis	*nifN*	✓	✓	✓
FeMoCo cofactor biosynthesis	*nifB*	✓	✓	✓
FeMoCo cofactor biosynthesis	*nifQ*	✓	✓	✓
Putative FeMoCo cofactor biosynthesis	*nifX*	✓	✓	✓
Putative FeMoCo cofactor biosynthesis	*nifT*	✓	✗	✓
Processing of FeMo protein	*nifU*	✓	✓	✓
Processing of FeMo protein	*nifS*	✓	✓	✓
Processing of FeMo protein	*nifY*	✓	✗	✗

Required for full activity of FeMo protein	*nifW*	✓	✓	✓
Required for full activity of FeMo protein	*nifZ*	✓	✓	✓
FeMo cofactor precursor	*nifO*	✓	✓	✓
FeMo cofactor carrier	*nafY*	✓	✓	✓

Supplementary Table S3. Percent Similarities for the Minimum Set of *nif* genes (*nifHADKENB*) required to perform nitrogen fixation among iron-oxidizing; *nifD* was most conserved between isolates in ZOTU11.

**16S rRNA % Similarity to O1**	**FeOB Isolate**	**Nitrogenase Subunit % Similarity to O1**						
		nifH	nifA	nifD	nifK	nifE	nifN	nifB
99.64	*Mariprofundus ferrooxydans* M34	100	99.43	100	100	99.15	100	99.8
96.50	*Mariprofundus* sp. EKF-M39	89.16	81.14	90.63	87.57	88.63	78.29	87.45

2 Supplementary Figures

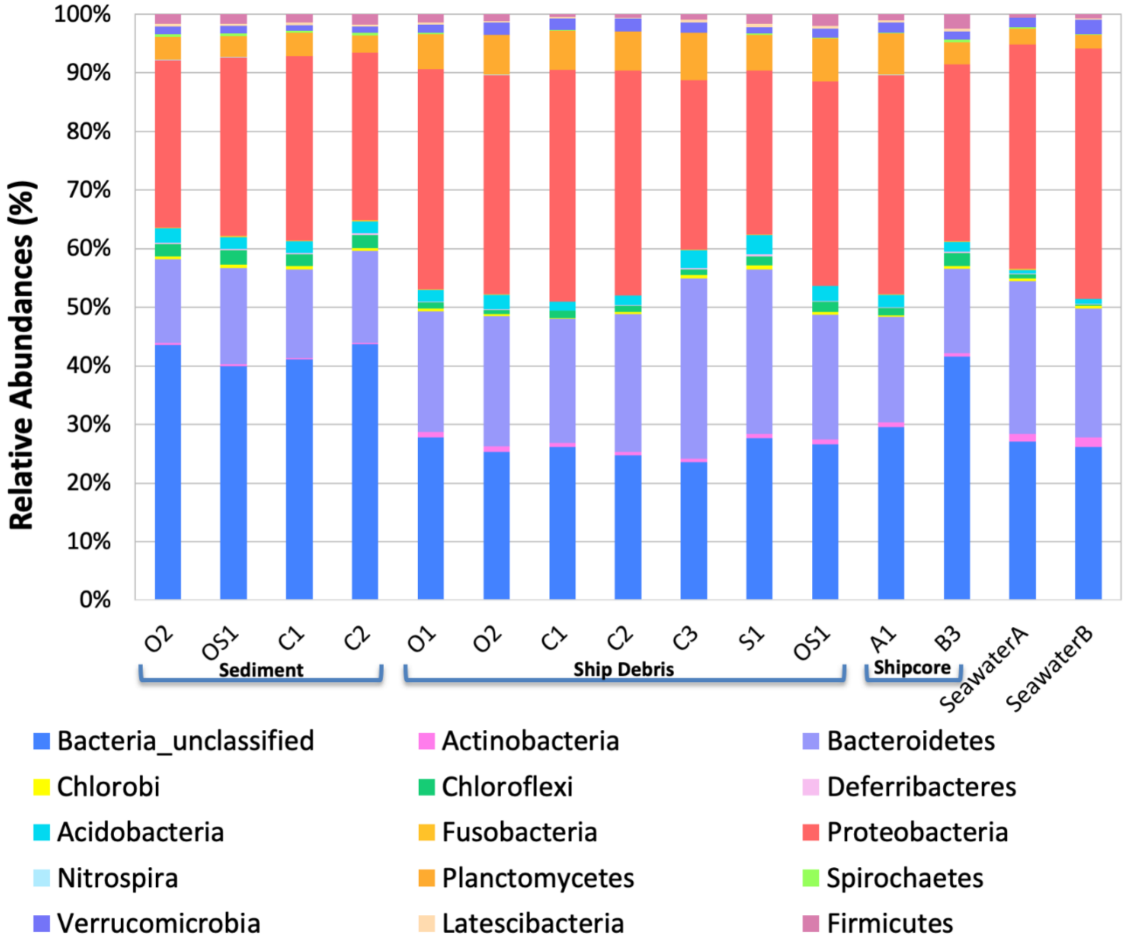

Supplementary Figure S1. Phylum level taxa plot of the shipwreck and surrounding environment microbial community composition present on each sample. Relative abundance calculated based on the percent of total phyla-level OTUs per sample.

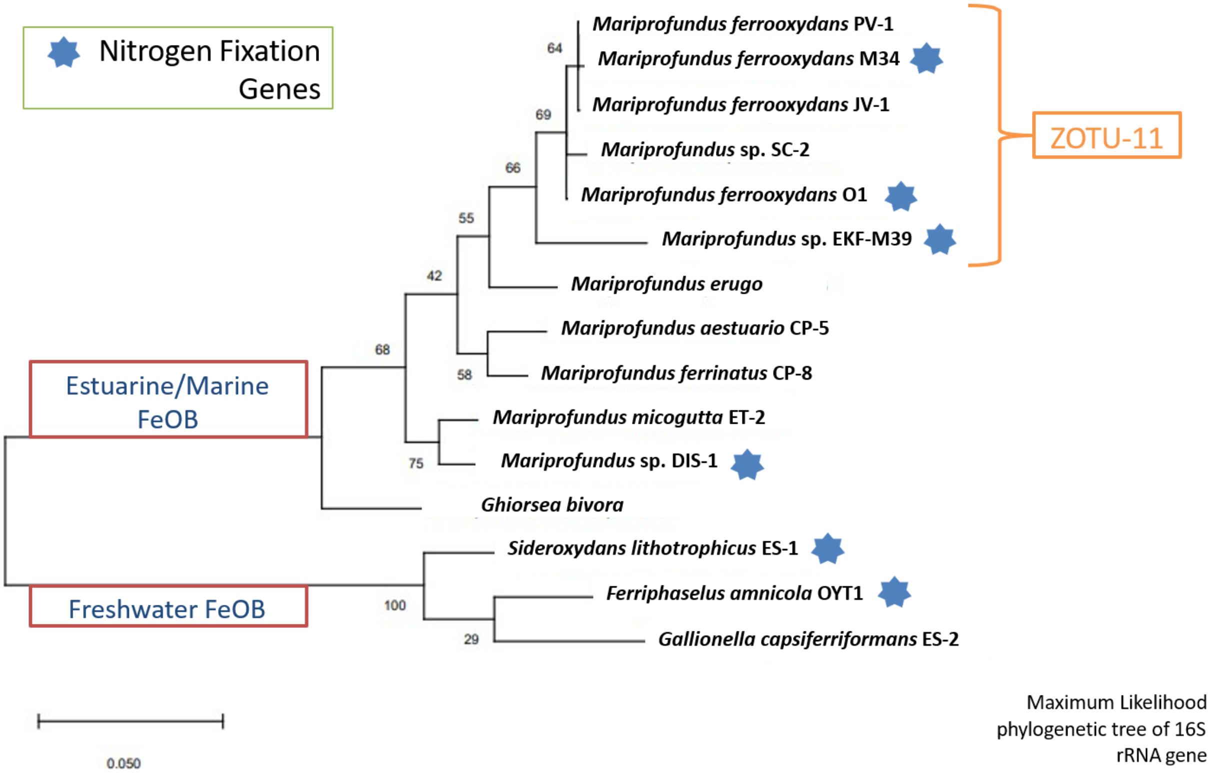

Supplementary Figure S2. Maximum likelihood phylogenetic tree showing *Mariprofundus ferrooxydans* O1 and the most closely related iron-oxidizers based on 16S rRNA gene comparisons. Numbers on branches indicate bootstrap values. Blue stars represent the presence of nitrogen fixation genes found within that isolate's genome. Nitrogen fixation capabilities have been confirmed in *Mariprofundus* sp. EKF-M39 and *M. ferrooxydans* M34.
